# Analysis of the vibration characteristics of tail beam of the hydraulic support under random coal gangue particle slip conditions

**DOI:** 10.1371/journal.pone.0269865

**Published:** 2022-06-10

**Authors:** Lirong Wan, Jiantao Wang, Dejian Ma, Qingliang Zeng, Zhaoji Li, Yang Yang

**Affiliations:** 1 College of Mechanical and Electronic Engineering, Shandong University of Science and Technology, Qingdao, China; 2 College of Information Science and Engineering, Shandong Normal University, Jinan, China; University of Genova, ITALY

## Abstract

To study the influence of the gangue content of coal gangue particles on the vibration signal of the tail beam under sliding condition, this paper combines three-dimensional(3D) laser scanning technology with the finite element method, establishes a finite element model of the real shape of coal gangue particles and the hydraulic support in top coal caving in LS-DYNA, analyzes the influence of gangue content on some characteristics of the acceleration signal on the tail beam in the time and frequency domains, and then studies the influence of the size and total mass of the rock, and the angle of the tail beam on the characteristics. The following conclusions are obtained: when the coal gangue particles slip on the tail beam, an increase in gangue content significantly improves the effective value of the acceleration signal of the tail beam in the time domain and the average power and average amplitude in the frequency domain. With different sizes, total masses, and tail beam angles, the increase in gangue content always causes an increase in acceleration signal characteristics. In terms of the influence of various factors on the same gangue content, at the same total mass, the larger the rock mass size is, the faster the characteristic value increases with the increase in gangue content. The greater the total mass, the greater the value of the acceleration signal characteristics. A smaller angle between the tail beam and the ground increases the value of each characteristic. The results of this study provide a reference for further research on coal gangue identification based on vibrations.

## 1. Introduction

Coal is the main energy source of China, the world’s largest energy producer, and consumer [1], and this situation will not change for a long time in the future [2]. In the major coal-producing countries in the world, thick coal seams account for a large part of the coal that is available for mining. The top coal caving method has become one of the main methods of thick coal seam mining because of its high yield and high efficiency [3–9]. It uses the comprehensive mechanical method to extract coal at the bottom of the coal seam, and then the top coal is broken under the action of ground pressure. It is released and transported through a special coal caving mechanism. The closing time of the coal opening affects the yield and quality of coal. When the coal is closed earlier, a large number of coal blocks are not released. When the coal is closed later, gangue will be mixed with coal and the quality of coal will be reduced. However, at present, the automation level of the drawing process of coal on the working surface in top coal caving is still relatively low, mainly relying on the underground workers to decide the time of closing by audio or visual observation. This method does not conform to the development trend of the automation of top coal caving. Therefore, the study of intelligent coal gangue identification technology is of great significance to current top coal caving method.

In recent years, many scholars have carried out research on coal gangue identification technology, and they have continued to find new methods to improve the accuracy and reliability of coal gangue identification. Zhang et al. proposed using natural gamma-ray technology to detect the instantaneous gangue content of coal gangue mixtures during top coal caving [10]. To solve the difficult feature extraction and poor feature credibility problems of coal and gangue, Guo et al. combined the difference in dielectric properties of coal and gangue with a support vector machine and proposed a coal gangue identification method [11]. Hou et al. proposed a method combining image feature extraction and an artificial neural network to identify coal and gangue through a coal gangue sorting system based on the difference in the surface texture and gray features of coal gangue [12]. Zou et al. combined near-infrared spectroscopy with improved generalized learning to develop software for coal gangue identification [13]. Wang et al. proposed a method combining dynamic weighing technology and three-dimensional laser scanning technology to detect the content of gangue by calculating the change in density [14]. Wang et al. used variational mode decomposition to reduce the noise of the response signal to obtain dielectric characteristics, and then proposed a coal gangue identification method based on the dielectric characteristics and geometric characteristics [15]. Liu et al. introduced the multifractal detrended fluctuation analysis method to extract the geometric characteristics of coal gangue, which improved the recognition rate of coal and gangue [16]. Lv et al. used group convolution to construct a new convolutional neural network as the backbone of the detector. When the detector was used for image recognition of coal gangue, the ability of the detector improved by 10% compared with that of a traditional detector [17]. The environment of a coal mining face has large water mist, high dust concentration, and high noise, these factors will seriously affect the effect of these coal gangue identification technologies [18–20]. However, a coal gangue identification method based on vibrations relies on the differences in the physical properties of coal and gangue and the contact characteristics of coal gangue and the tail beam, so it can effectively prevent the interference of many factors and has great development potential [18].

To study the difference in the vibration signals of the tail beam after coal and gangue contact with the tail beam, Zeng et al. carried out a dynamic analysis of coal and gangue particle impact shield beams under different collision conditions and studied the influence of the rock shape, velocity, and rock material on the impact process [21]. Wan et al. simulated the dynamic process of coal rock particles impacting tail beams at different positions and different coal caving angles and found that the dynamic response and collision contact force of tail beam are different at different positions [22]. After simulating the process of rock impacting a metal plate with the same mass and different shapes, Yin et al. found that different shapes of rock produced different vibration signals for the metal plate [23]. Researchers have studied the difference in the vibrations of hydraulic supports after impact by coal and gangue, but most of researchers have assumed the particles to have an ideal shape without considering the real shape of particles.

The shape effect of rock has been widely studied. Yao et al. studied the influence of gravel content and gravel shape in a soil-rock mixture on its shear performance and found that the shape of gravel can affect its shear performance more than the gravel content [24]. Liang et al. found that the elastic modulus increased with increasing specimen size, and the fracture mode was closely related to the shape of the specimen when testing the mechanical properties of rocks [25]. Das et al. used smoothed particle hydrodynamics (SPH) to simulate the uniaxial compression process of rock and found that rock shape had a great influence on the fracture process and debris shape [26]. Lu et al. used the discrete element method to analyze the stability of a clay-rock mixture slope and found that the shape of gravel had an impact on the stability of the slope [27]. Because the shape of rock has an important influence on its impact characteristics, damage characteristics, etc. and these characteristics affect the vibration signal, it is necessary to use the real particle shape to verify the reliability of identifying the properties of particles through the vibration signal.

**Fig 1:**
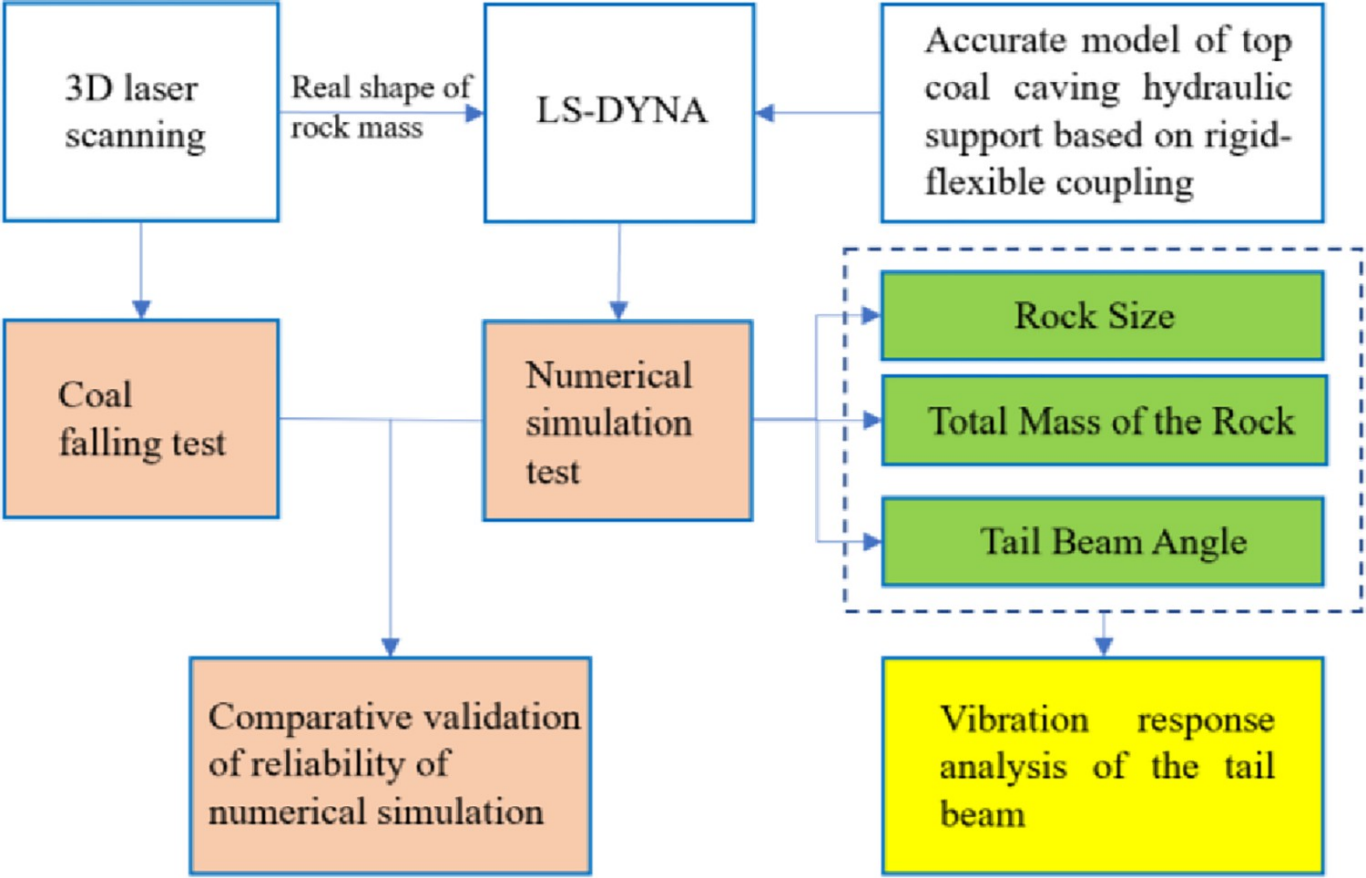
Working process of this study.

By analyzing the existing research on coal gangue identification based on vibrations, the contact mechanics between coal rock and metal plates and the vibration difference principle have been studied in depth. However, these studies assume that the shape of a rock mass is ideal when simulating contact between coal gangue and a metal plate. Although this will simplify the problem, the influence of rock shape is ignored, and the shape effect of rock mass has been studied in other fields. Therefore, it is necessary to verify the identifiability of the gangue content of coal gangue particles by considering a real coal gangue particle shape. At the same time, most scholars have only studied the instantaneous process of coal gangue particles impacting the tail beam and have paid little attention to the process of coal gangue particles sliding along the tail beam; however, sliding is the most likely movement form of coal gangue particles in the process of coal caving. Therefore, this paper uses 3D laser scanning equipment to obtain the shape information of coal blocks and then establishes a finite element model of coal gangue and hydraulic support using LS-DYNA software. The process of coal gangue particles sliding along the tail beam is analyzed, and the influence law of the total mass of rock, rock mass size, and tail beam angle on the vibration signal is given. A schematic of this study is shown in Fig 1.

The contribution of this paper to existing coal gangue identification research based on vibrations are as follows:

1. The sliding process of coal gangue particles with real shapes along the tail beam is simulated.
2. The characteristics of vibration signals in the frequency domain are analyzed, and the regularity between the average power and average amplitude of gangue content and acceleration signals in the frequency domain is found.
3. The influence of the tail beam angle and the size and total mass of the rock mass is analyzed. It is proven that the method of identifying the change in the gangue content by monitoring some characteristics of the vibration signal of the tail beam has good adaptability.

The following sections of this paper are organized as follows. Section 2 introduces the finite element model of the real rock shape and hydraulic support and tests the constitutive model of coal. Section 3 simulates the process of eight coal gangue particles sliding along the tail beam and then analyzes the influence of the rock mass size, total mass, and tail beam angle. Section 4 presents the conclusion.

## 2. Models and methods

It is difficult to understand the influence of coal gangue particles on the vibrations of the hydraulic support through experiments because so many factors affect the vibration response of the support. However, numerical simulations are is a reliable and powerful method used to simulate the vibrations of tail beams. In this paper, LS-DYNA software is used to simulate the process of coal gangue particles sliding on the tail beam. In this section, a numerical simulation method is presented to study the vibration response of the tail beam when coal gangue particles are slipping. First, finite element models of top coal caving hydraulic support and real shape coal gangue particles are established, and each part is assigned to the material model to reflect the material behavior under impact. The contact algorithm between the rock mass and tail beam, and the rock mass and rock mass are defined in LS-DYNA. Finally, the constitutive model parameters of coal materials are verified by testing.

### 2.1 Reconstruction of the real shape rock model

**Fig 2:**
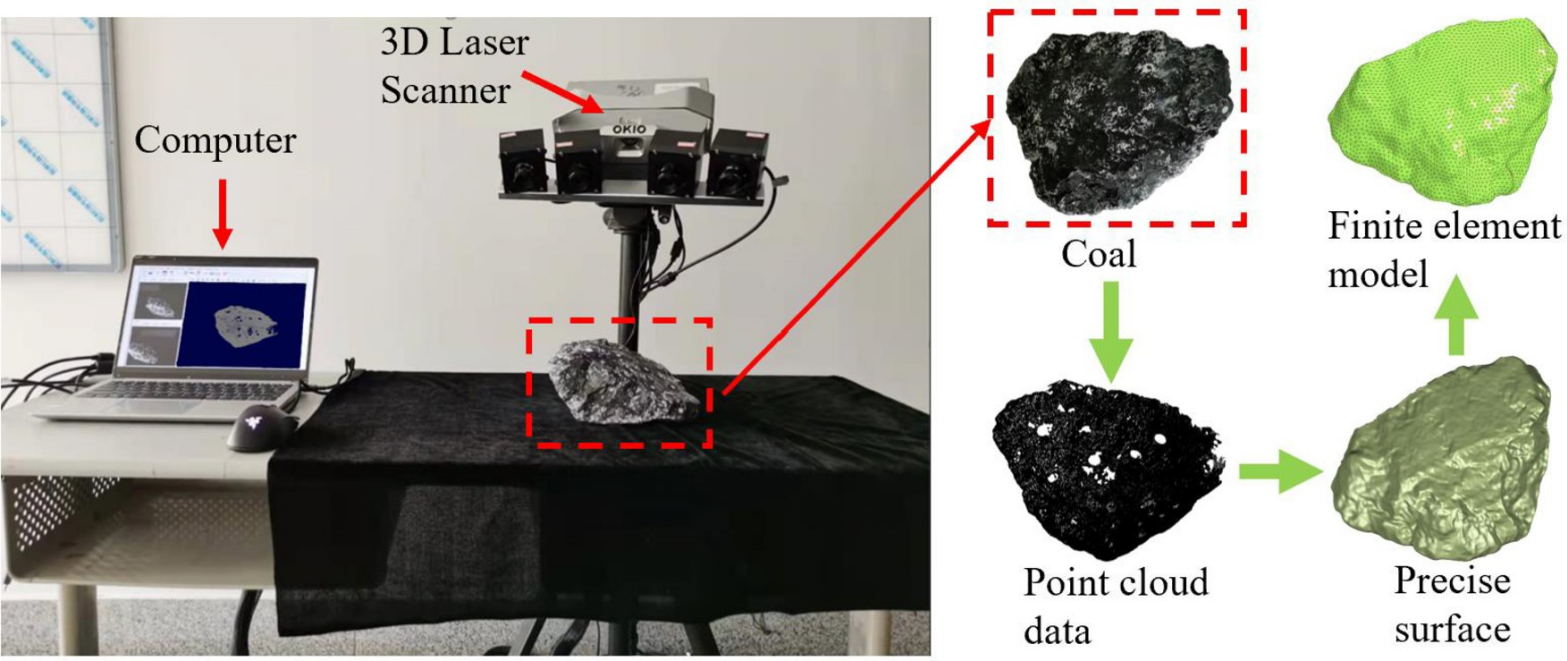
The process of modeling the coal block.

The 3D laser scanner is used to obtain 3D point cloud data of the coal block surface. Fig 2 shows the process of modeling the coal block. After scanning, the point cloud data of a part of the shape of the rock particles are transmitted to the software running on the computer, and the software automatically splices the data from each scan. In the software, the point cloud data obtained can be observed in real time, and the complete 3D model can be obtained by continuously changing the angle of the coal block. Then, the point cloud data are processed into an accurate surface using the postprocessing software Geomagic Studio. Finally, a finite element model is generated in HyperMesh according to the contour of the accurate surface, and the element type is tetrahedral.

### 2.2 Establishment of hydraulic support model

**Fig 3:**
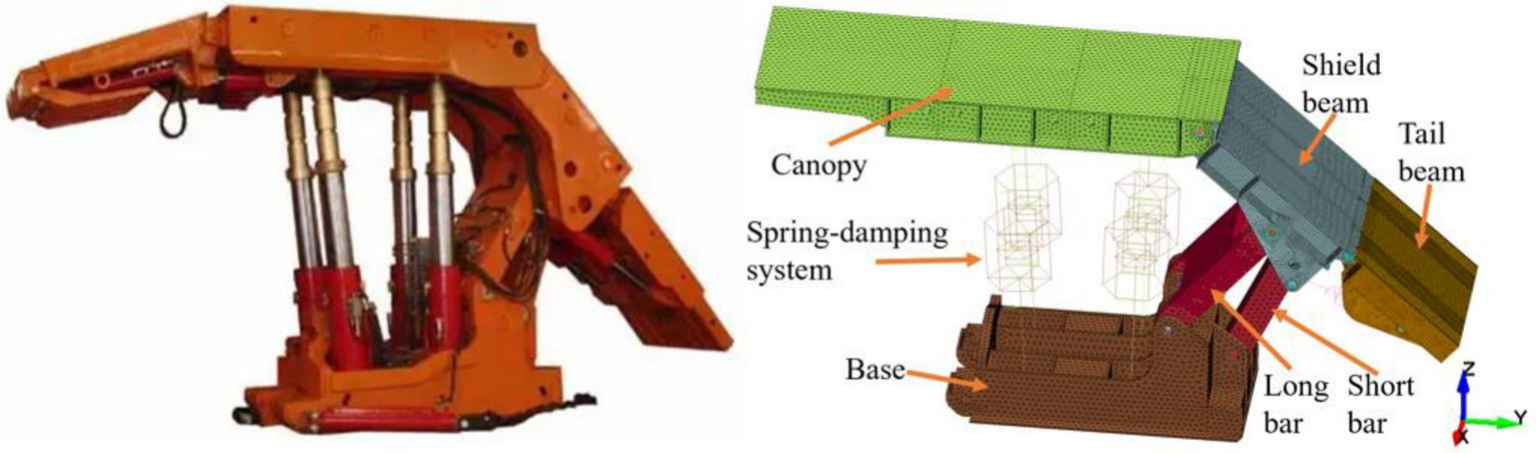
The hydraulic support. For physical device (left) and finite element model (right).

With a 1:1 ratio, a rigid-flexible coupling model of a ZF4800-17-32 top coal caving hydraulic support is established. The maximum and minimum working heights of the hydraulic support are 3.2 meters and 1.7 meters, Respectively. The telescopic beam and the insert plate are omitted because they have little effect on the vibration response of the tail beam. The shield beam is parallel to the tail beam, and its angle with the ground is 40°. HyperMesh is used to mesh the hydraulic support model. To improve the accuracy of the calculation results, hexahedral elements with higher calculation accuracy are used on the tail beam and shield beam, and tetrahedral elements are used in other parts. The total number of elements of the hydraulic support is 233472. The rotational connection between the parts of the hydraulic support is realized by creating a virtual rotational pair, and a spring-damping system is used to replace the hydraulic cylinder. The physical device and rigid–flexible coupling model of a top coal caving hydraulic support is shown in Fig 3.

Ls-PrePost is used to preprocessing of the model. The freedom of the nodes at the bottom layer of the base is constrained, and the acceleration is set to 9.8 m/s^2^ to simulate the effect of gravity. The contact between the rock mass and the tail beam is defined by CONTACT_AUTOMATIC_SURFACE_TO_SURFACE, and the contact between the rock mass and the rock mass is defined by CONTACT_AUTOMATIC_GENERAL. The main structure of hydraulic support is made of high strength steel, such as Q345, and Q460. The material model of the steel plate is the Piecewise Linear Plasticity, and the parameters of the model are shown in Table 1 [28]. MAT_JOHNSON_HOLMQUIST_CONCRETE (HJC) is selected as the material model of coal and gangue. The HJC model can simulate the compression damage behavior of rock after impact [29]. The HJC model contains 21 parameters, including basic mechanical parameters *f*_c_, *G*, *T* and *ρ*; strength parameters *A*, *B*, *N*, *S*_max_ and *C*; damage parameters D_1_, D_2_, *ε*_*f*min_; pressure parameters *K*_1_, *K*_2_, *K*_3_, *p*_1_, *μ*_l_, *μ*_c_; reference strain rate *έ*_0_ and failure type *f*_s_. Details of the model can be found in reference [30]. The failure parameter *f*_*c*_ of the HJC model cannot simulate the failure of materials, so the keyword MAT_ADD_EROSION is needed to simulate the fracture of materials. Because the mechanical properties of coal and rock are similar, the HJC model is used as the material model of coal and gangue [31, 32]. The physical properties of gangue are similar to those of granite. The HJC model parameters of gangue are shown in Table 2 [33]. Long et al. divided the parameters of the HJC model into sensitive parameters and insensitive parameters [34], according to the parameter sensitivity analysis of HJC model in his study, *A*, *B*, *G*, *p*_l_, *μ*_l_, and *f*_c_ are determined as sensitivity parameters, When the sensitivity parameters and insensitivity parameters change with the same amplitude (±20%), the influence of sensitivity parameters on the test results is greater than that of insensitivity parameters. Some parameters in this paper refer to citation [35], and then, according to the falling coal test in section 2.3.1, we continuously adjust the parameters used in the numerical simulation in section 2.3.2, and finally obtain the numerical simulation results consistent with the test. The HJC model parameters of coal are shown in Table 3.

**Table 1:** Material model parameters of metal plate.

$\rho_{\left(\mathrm{kg}/m^{3}\right)}$	E(Pa)	u	σ_0_(Pa)
7830	2.07e11	0.3	2.07e8
C	P	ε_f_	
40	5	0.75	

**Table 2:** HJC material model parameters of gangue.

ρ_0_(kg/m^3^)	G(Pa)	f_c_(Pa)	C	N	S_max_	D_1_	D_2_	ε_fmin_
2590	1.114e10	1.223e8	0.006	0.6	7.7	0.04	1	0.01
T(Pa)	p_c_(Pa)	μ_c_	p_1_(Pa)	μ_1_	k_1_	k_2_	k_3_	f_s_
7.76e6	4.075e7	0.0023	1.65e9	0.11	1.15e10	2.6e10	5e10	0

**Table 3:** HJC material model parameters of coal.

ρ_0_(kg/m^3^)	G(Pa)	f_c_(Pa)	C	N	S_max_	D_1_	D_2_	ε_fmin_
1400	5.8e8	9e6	0.006	0.76	7.7	5e-6	1	2e-6
T(Pa)	p_c_(Pa)	μ_c_	p_1_(Pa)	μ_1_	k_1_	k_2_	k_3_	f_s_
1.86e6	3e6	8e-4	3.4e8	0.1	1.6e9	1.7e8	5.8e9	1.4

### 2.3 Verification of the coal material model

The strength of coal is far less than that of gangue, so coal is more likely to break when contacting the tail beam, which affects the vibration signal on the tail beam. To ensure the accuracy and reliability of the numerical model, the coal fragmentation processes in the real coal falling test and numerical simulation are compared.

#### 2.3.1 Coal falling test

**Fig 4:**
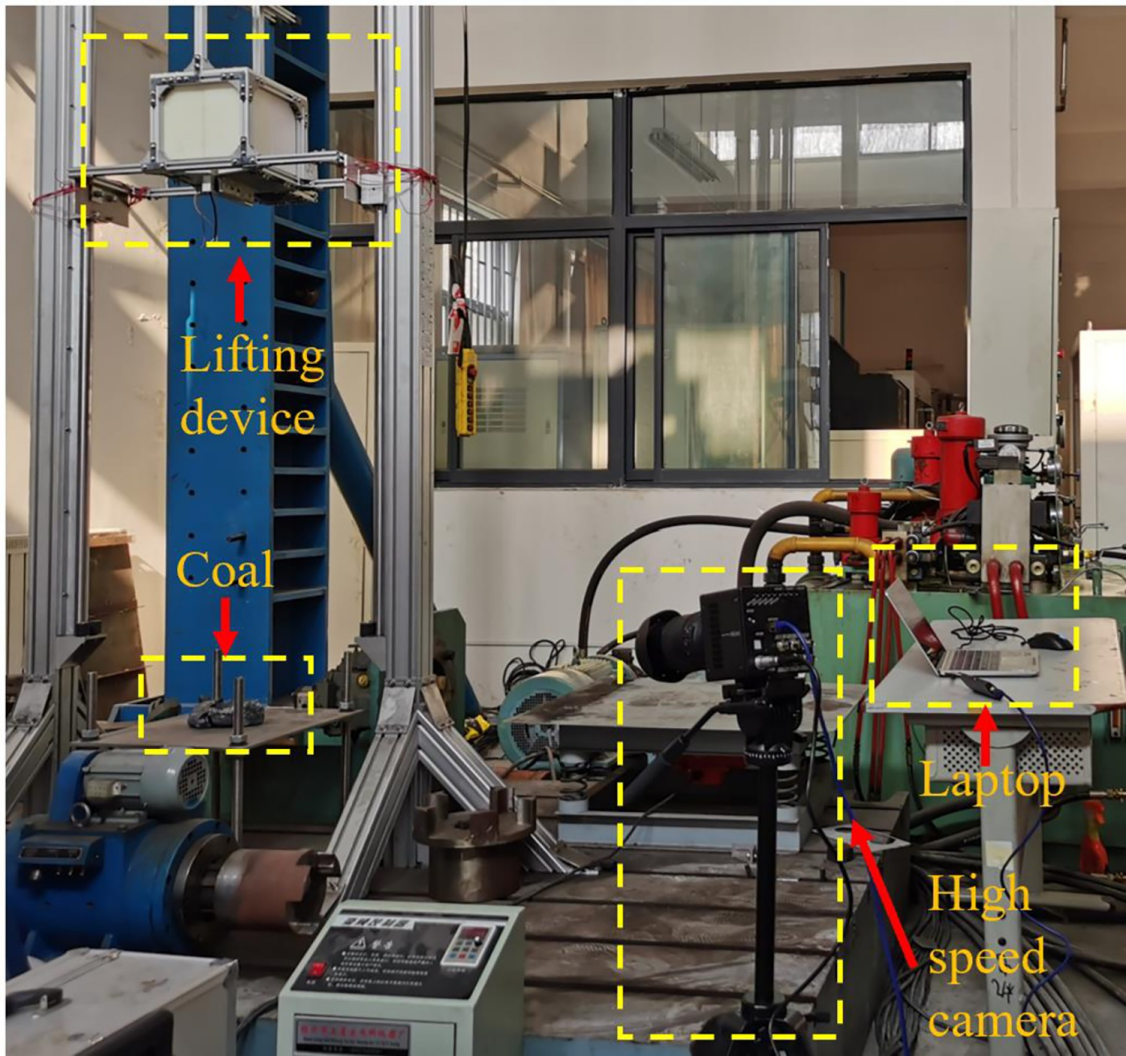
The coal falling test.

Fig 4 shows the process of the coal falling test. A 2.4 kg coal block is raised to 3.3 meters on the test bench by using a lifting device. A steel plate which is fixed around is below the coal block. The coal block is released and falls freely and impacts the steel plate. A high-speed camera is targeted at the steel plate to record the process of the coal block breaking after impacting the steel plate.

#### 2.3.2 Numerical simulation

**Fig 5:**
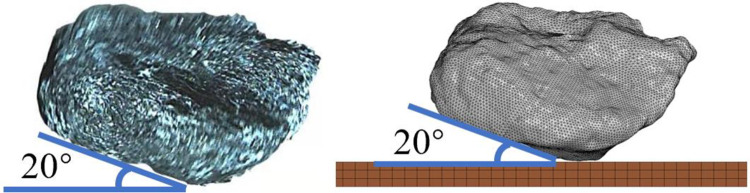
The finite element of coal falling test. For true coal block (left) and finite element model (right).

The finite element model of the coal falling test is shown in Fig 5. The falling attitude of the coal block is the same as that in the coal falling test, and the freedom of the nodes around the elements of the steel plate is constrained. Since the coal block falls from 3.3 m in the coal falling test, the speed of the coal block is set to 8 m/s, and the length, width, and height of the metal plate are 400 mm, 400 mm, and 15 mm, respectively. The material models and the contact settings are the same as above.

#### 2.3.3 Comparison of the results

**Fig 6:**
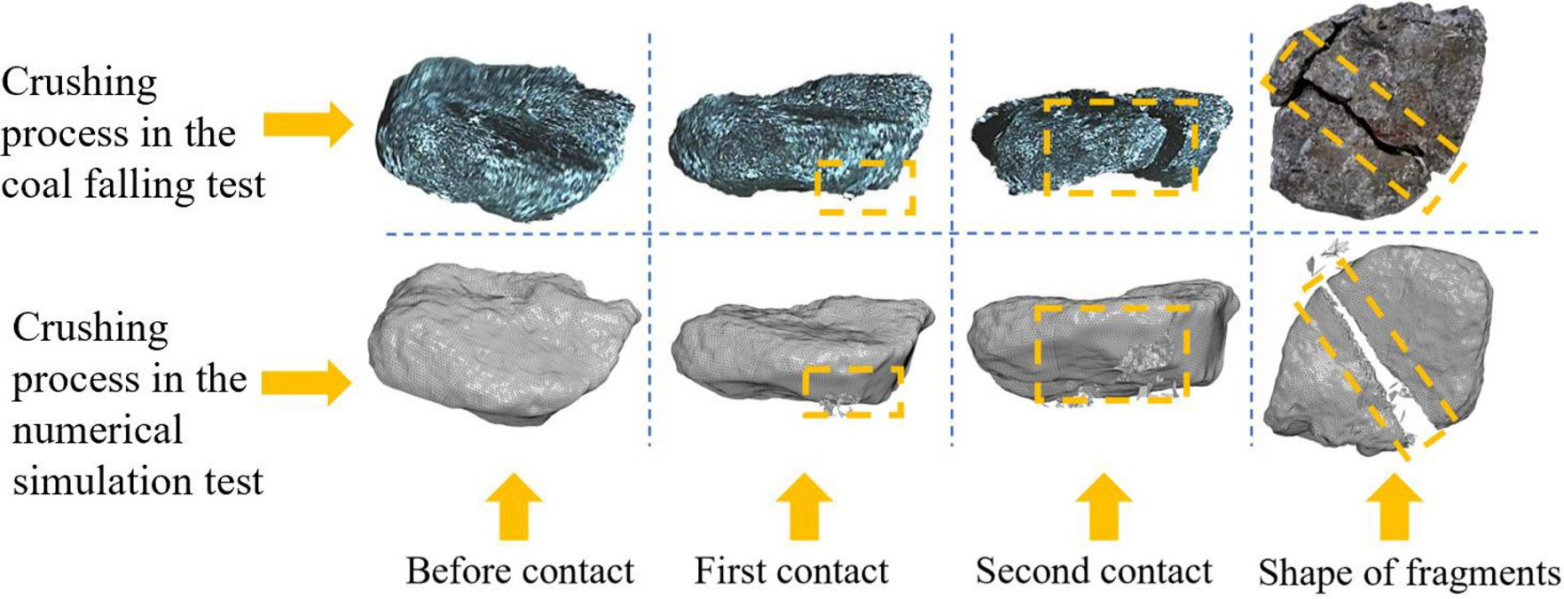
Comparison of the real test and numerical simulation.

Fig 6 shows a comparison of the coal fragmentation processes in the coal falling test and the numerical simulation. The posture of the coal block before and after the first and second contact with the steel plate and the final fragment shape are shown in the figure. When the coal block and the steel plate are first in contact, the coal block in the collision position produces some fragmentation, and the impact causes the defects and cracks in the coal block to expand. However, the coal block does not produce large size fragments. Due to its irregular shape, the coal block then flips in the air and contacts the steel plate for the second time. At the position of impact, some small fragments are produced, and the coal block breaks into two large fragments. The results of the coal falling test are consistent with the results of the numerical simulation, which proves that the constitutive model parameters of coal can simulate the real coal fragmentation process.

## 3. Results and analysis

When coal caving is carried out on the fully mechanized working face, the broken top coal is first directly discharged from the gap formed between the tail beam and the goaf, with the decrease in the gap size, the top coal gradually accumulates on the tail beam and the shield beam; When the coal opening is opened, the coal gangue particles slip down the upper surface of the shield beam and the tail beam under the action of gravity and fall on the scraper conveyor below. To study the influence of the gangue content of coal gangue particles on the vibration signal of the tail beam during the slipping process, this paper designs a numerical simulation test of coal gangue particles with different gangue contents slipping along the tail beam discusses the difference in the vibration signal in the time and frequency domains and then analyzes the influence of the size and total mass of the rock and the angle of the tail beam on the signal difference.

### 3.1 Influence of the gangue content on the vibration signal

**Fig 7:**
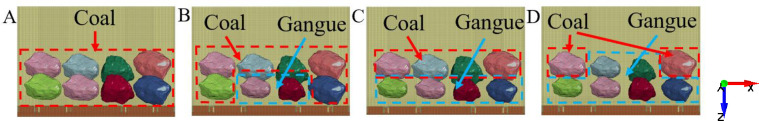
Four coal gangue particles slipping tests with different gangue content. (A) The gangue content is 0%. (B) The gangue content is 25%. (C) The gangue content is 50%. (D) The gangue content is 75%.

To study the influence of different gangue contents on the vibration signal of the tail beam in the sliding process, a series of numerical simulation tests are designed in this section as shown in Fig 7. Eight rock particles with the same mass are placed above the tail beam, and there is a minimum gap between the rock mass and the tail beam. The rock mass is only affected by gravity and the initial velocity is 0 m/s. The gangue contents are 0%, 25%, 50% and 75%. The simulation time is set to 0.5 s, and the output interval of the data is 0.005 s.

**Fig 8:**
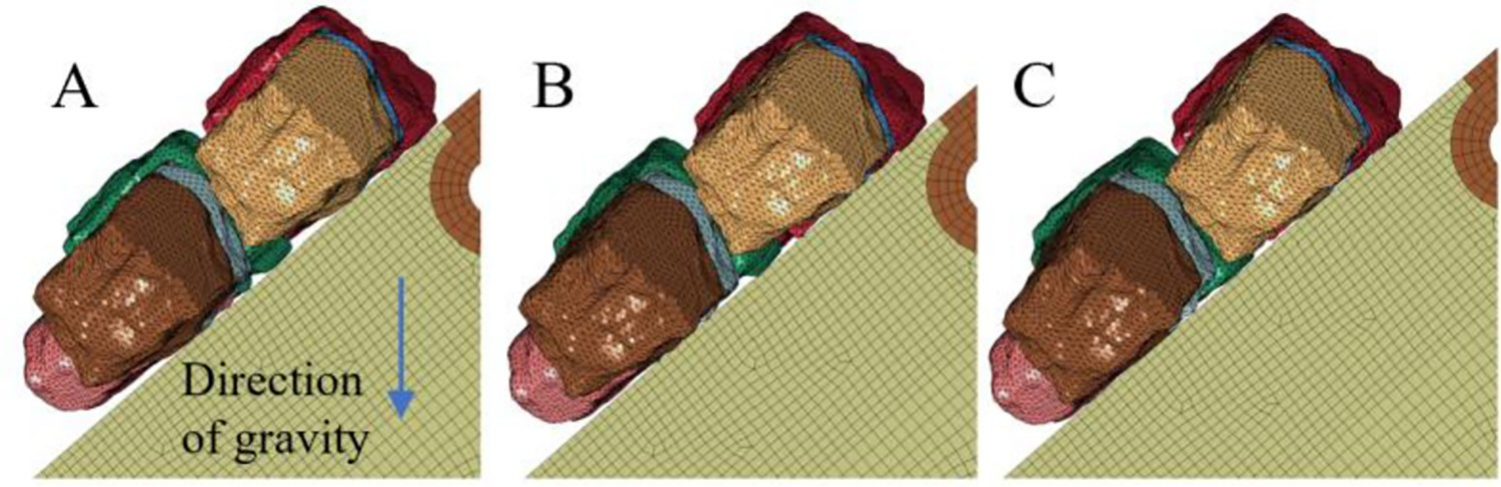
The falling process of the rock mass. (A) At the beginning of the simulation. (B) Rock mass impact the tail beam. (C) Rock mass begin to slip.

As shown in Fig 8, the numerical simulation shows that the rock mass falls under the action of gravity and has a slight impact on the tail beam, and then slips down along the tail beam. The motion state of the rock mass gradually stabilizes and has stable sliding contact with the tail beam.

**Fig 9:**
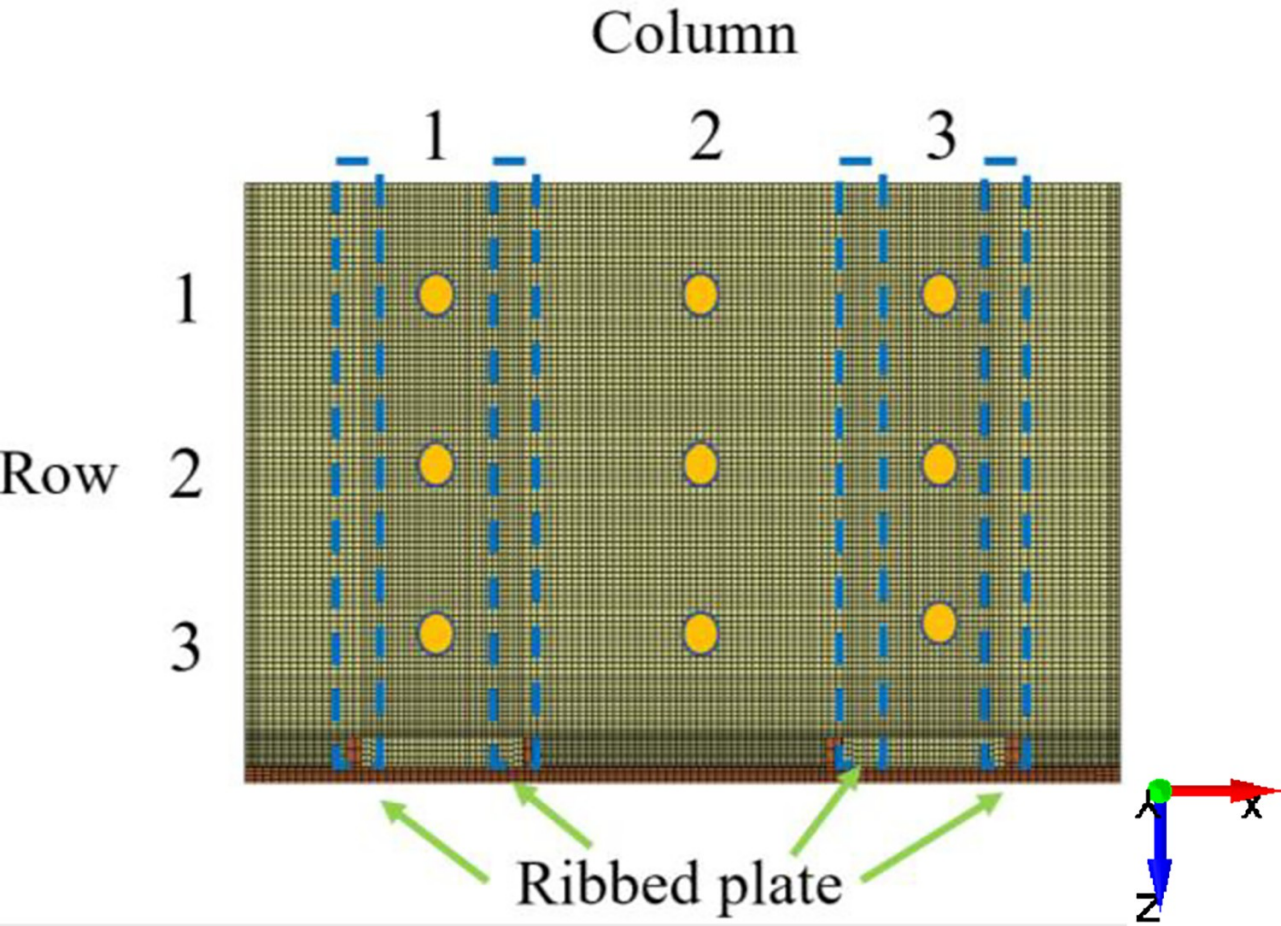
Nine nodes on the tail beam.

**Fig 10:**
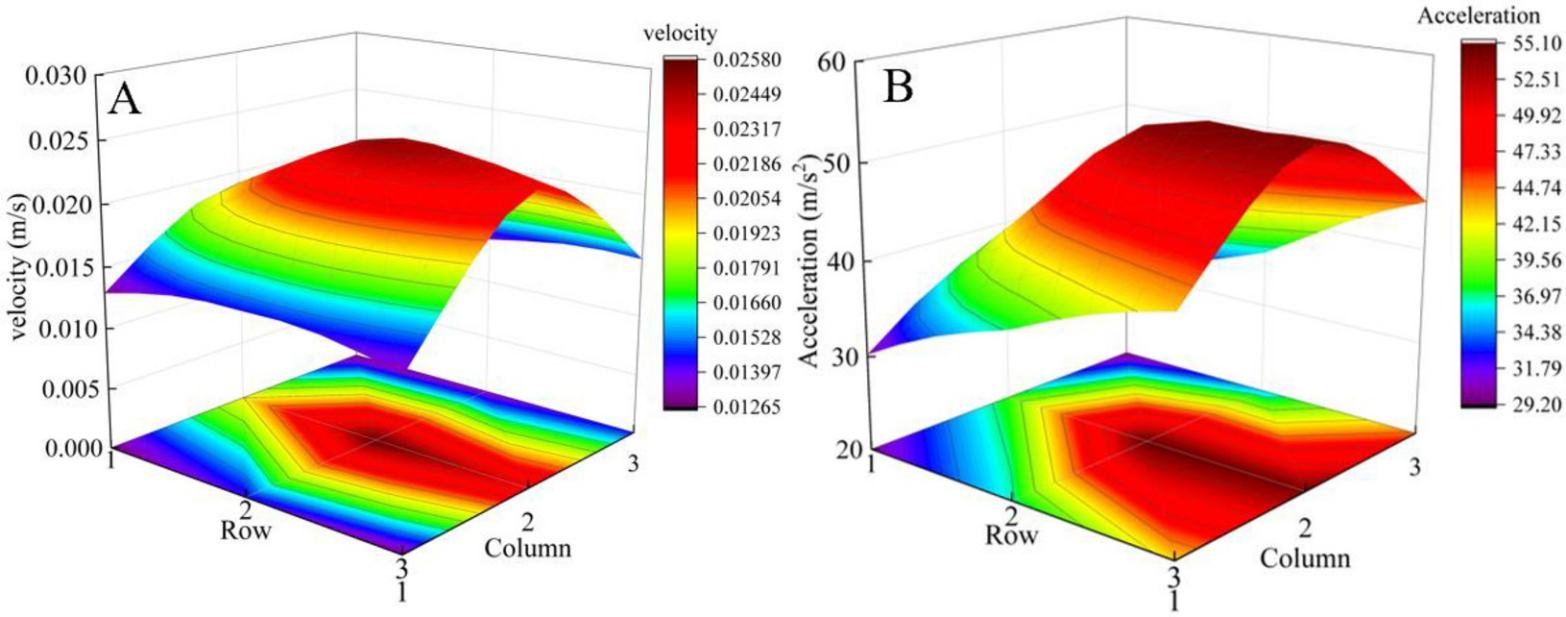
Strength distribution of vibration signals on tail beam. (A) The strength distribution of velocity signals. (B) The strength distribution of acceleration signals.

The experiment with a gangue content of 0% is selected to extract the velocity and acceleration signals of nine nodes on the tail beam, as shown in Fig 9, and to calculate the effective value. The intensity distribution of the velocity and acceleration signals on the tail beam is shown in Fig 10. From the direction of the row, a strong vibration signal appears in the third row because there is a small gap between the rock mass and the tail beam. When the numerical simulation starts, the rock mass begins to slide after impacting the tail beam, and the force generated by the impact process is greater than that generated by the slipping process. From the direction of the column, strong vibration signals are prone to appear in the second column because this area is far from the ribbed plates, which greatly limits the free vibration of the steel plate of the tail beam. This result provides a reference for the arrangement of sensors on the tail beam.

**Fig 11:**
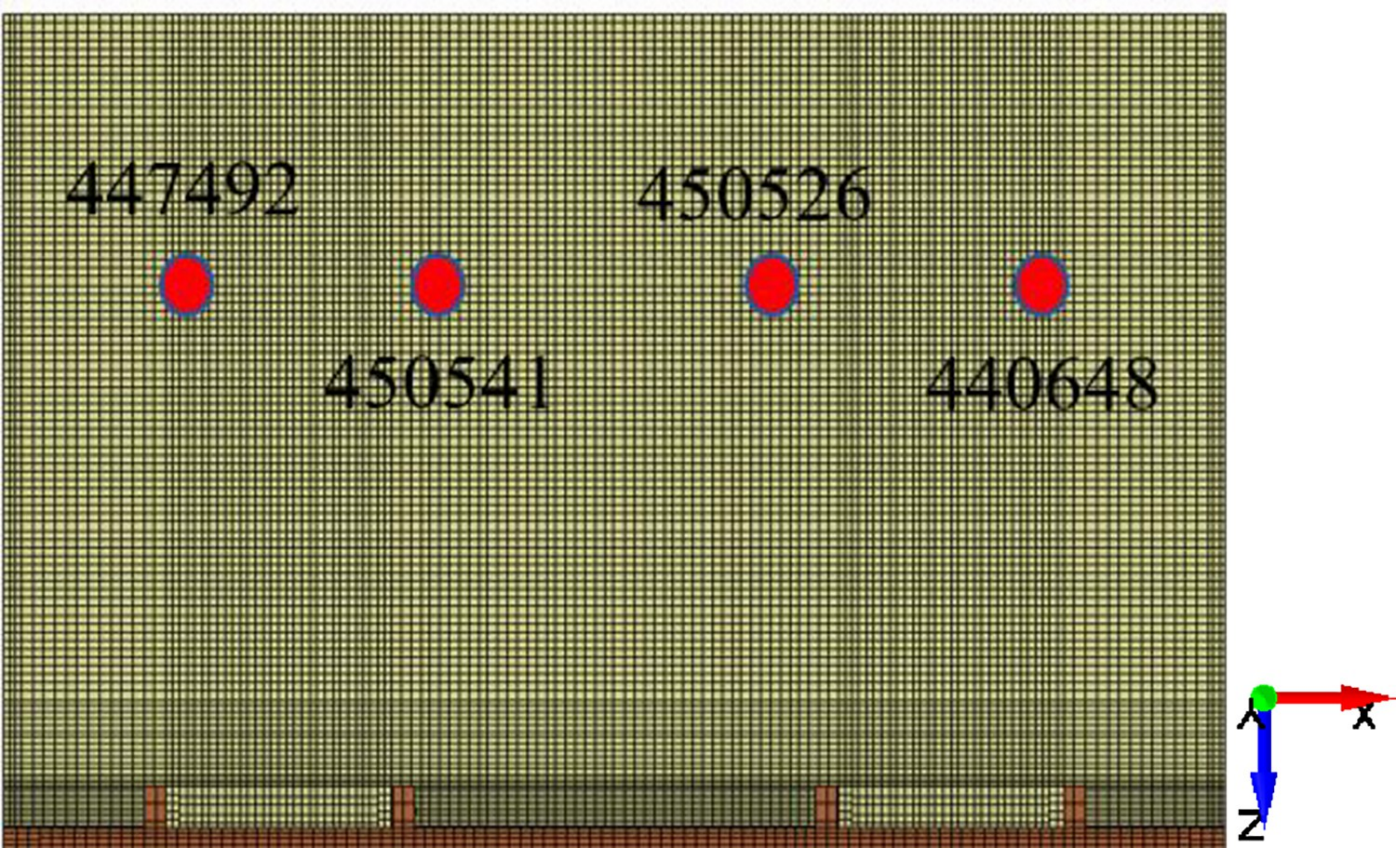
Four nodes on the tail beam.

**Fig 12:**
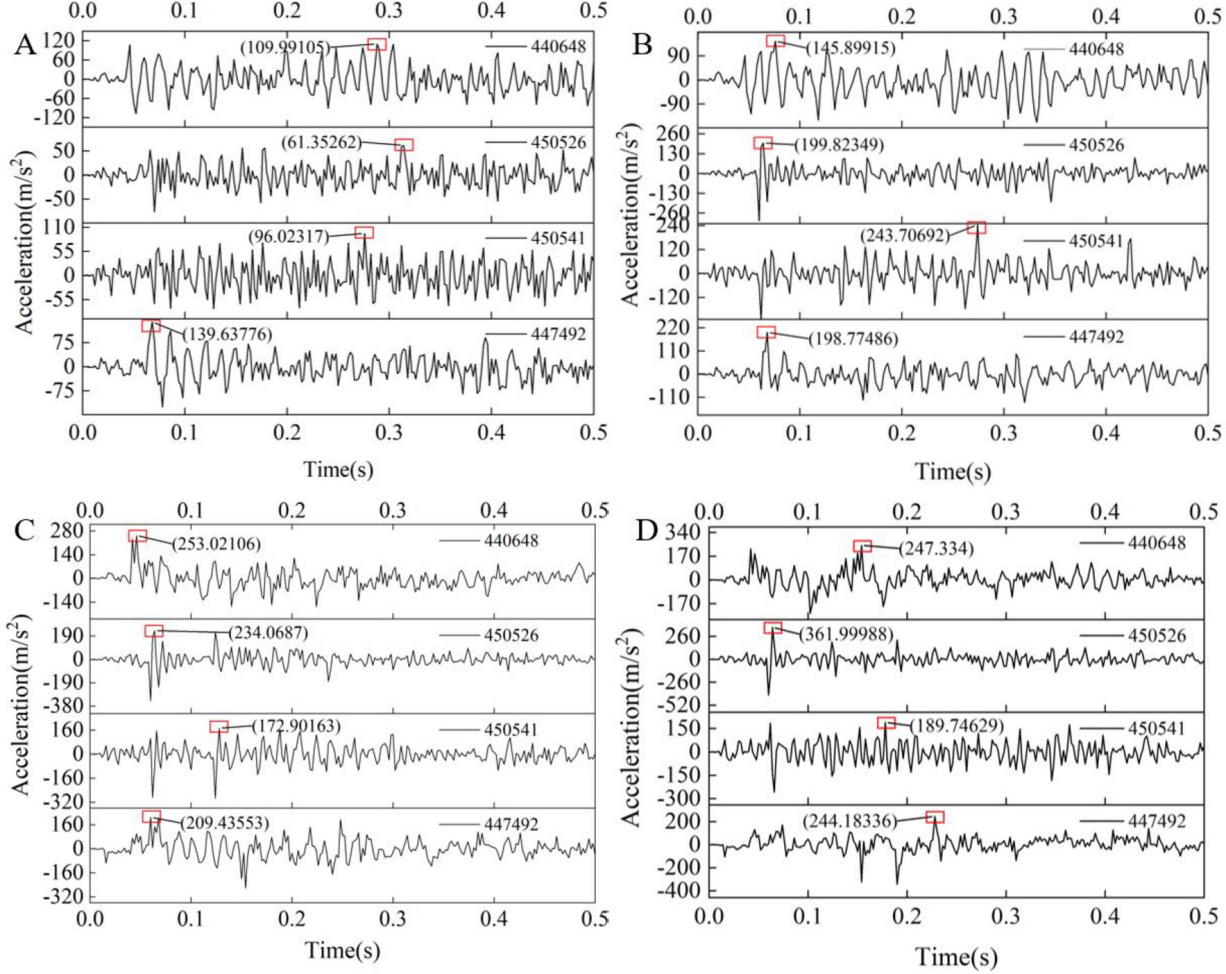
The acceleration signals of four nodes with different gangue content. (A)The gangue content of 0%. (B) The gangue content of 25%. (C) The gangue content of 50%. (D) The gangue content of 75%.

The nodes at four positions shown in Fig 11 are selected on the tail beam. The rock particles pass through these four nodes when they slide along the tail beam, and the acceleration signals perpendicular to the ground direction of each point are extracted. The acceleration signals of each group of tests are shown in Fig 12. The acceleration signal of 0.5 s contains 100 sampling points. By observing the maximum peak value of the acceleration signal, the maximum peak values of the signals of each node shows an upward trend with increasing gangue content. Taking node 440648 as an example, the maximum peak values are 109.99, 145.9, 253.02, and 247.33. Since the hardness characteristics of coal and gangue are quite different, gangue will produce relatively strong acceleration signals after contact with the tail beam.

The time domain characteristics of the acceleration signal are analyzed. The effective value can be used to describe the average energy of the signal, calculate the effective value of the acceleration signal of each node, and then calculate the average value of the four nodes. The calculation formula of the effective value is

$$X_{\mathrm{rms}}=\sqrt{\frac{\sum_{i=1}^{N}x_{i}^{2}}{N}}$$ (1)

where *X*_rms_ is the valid value, *x*_*i*_ is the value of the sampling point, and *N* is the number of sampling points.

**Fig 13:**
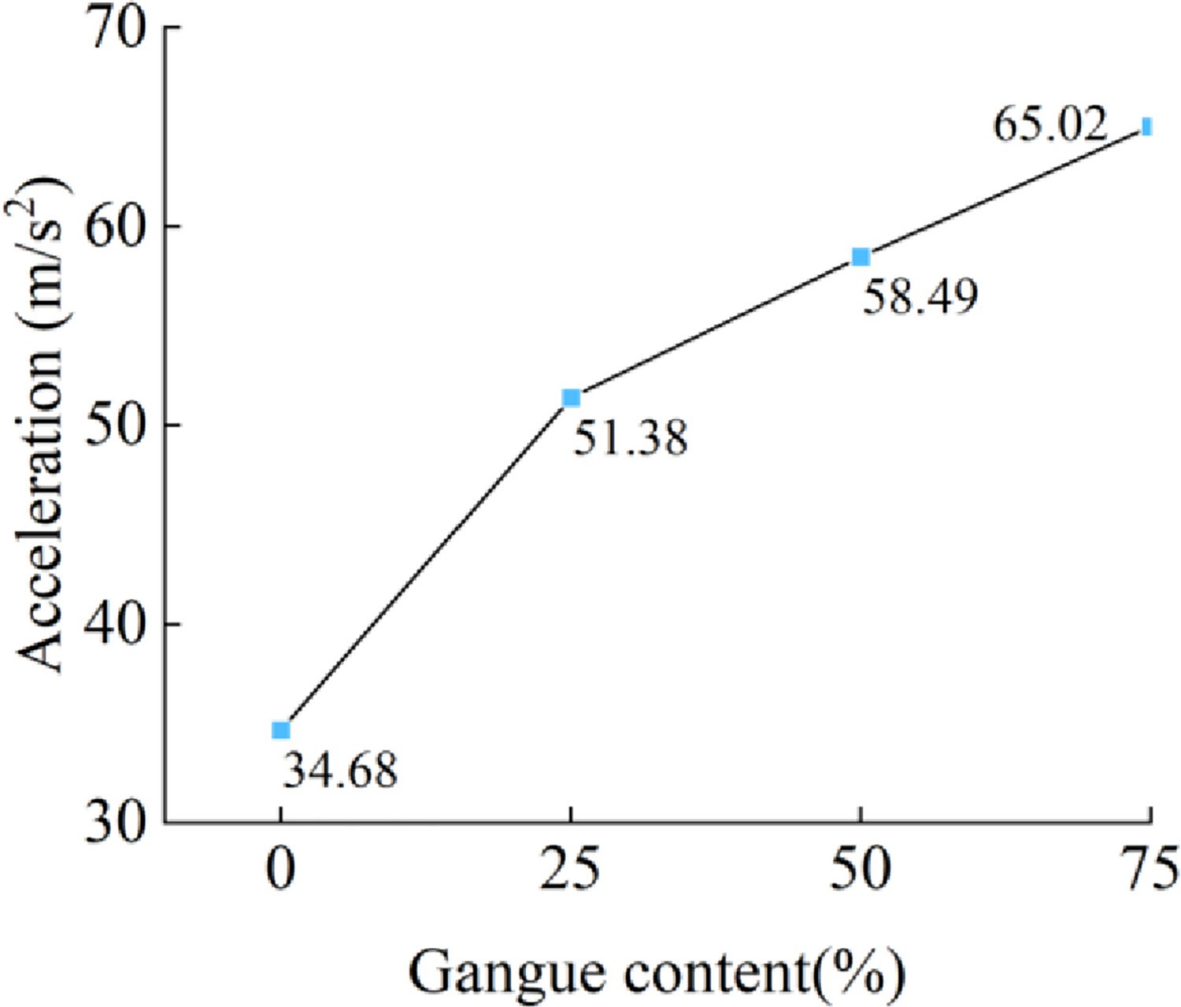
Gangue content versus the effective value of acceleration signal.

The results are shown in Fig 13. The changing trend of the effective value is similar to that of the maximum peak value, indicating that when the coal gangue particles are in sliding contact with the tail beam, the increase in the gangue content causes an increase in the average energy of the acceleration signal.

**Fig 14:**
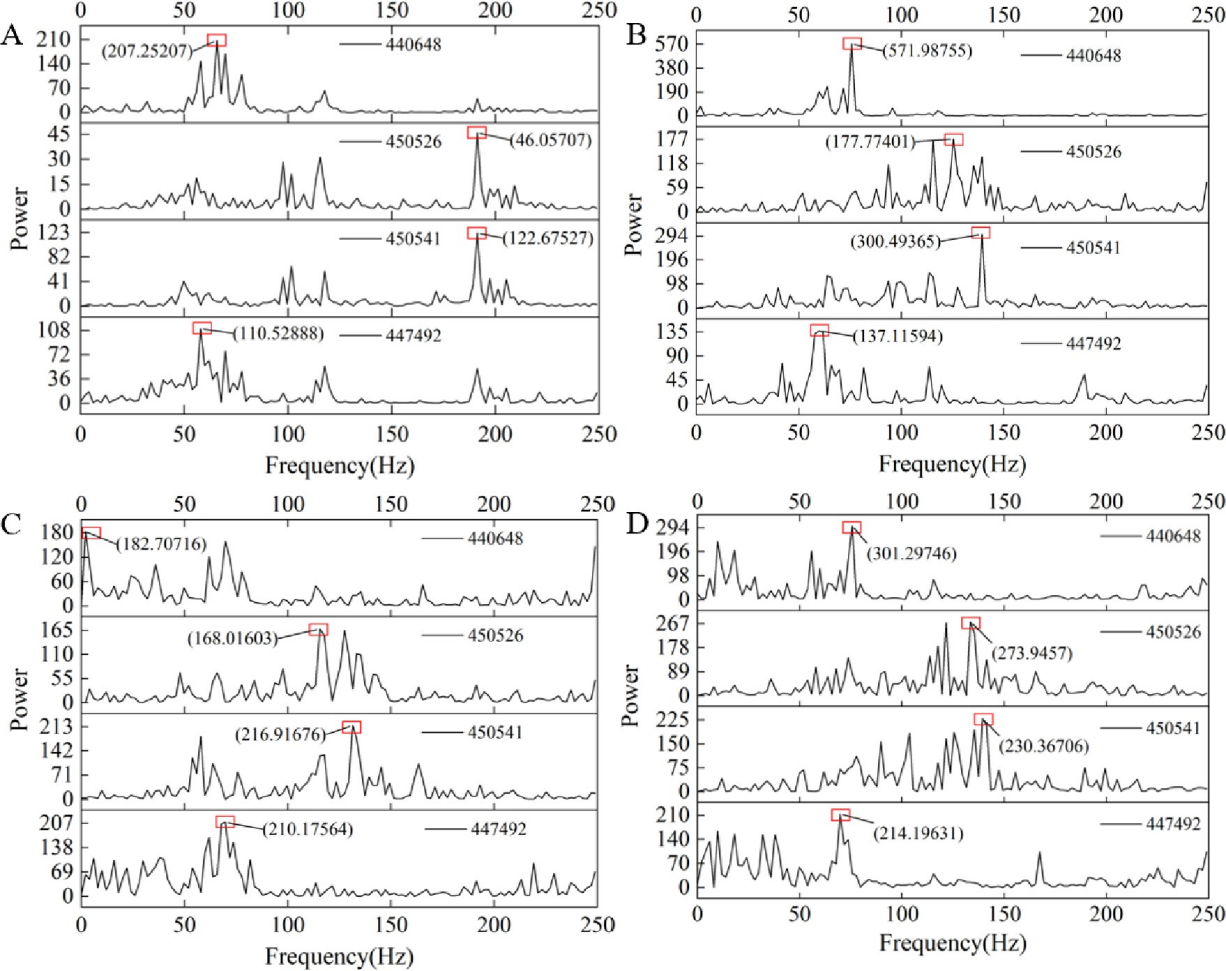
The power spectrum of four nodes with different gangue content. (A) The gangue content of 0%. (B) The gangue content of 25%. (C) The gangue content of 50%. (D) The gangue content of 75%.

**Fig 15:**
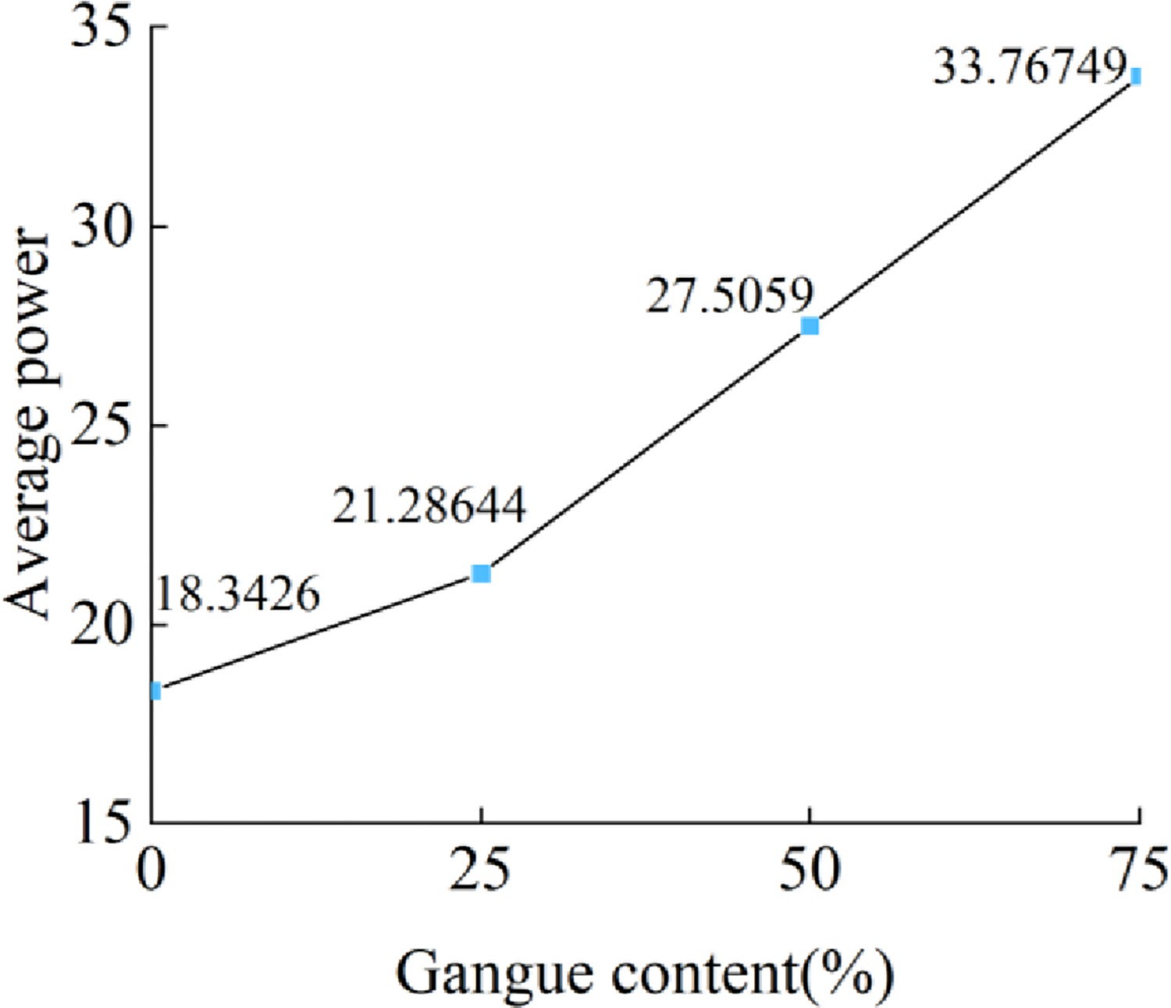
Gangue content versus the average value of the power.

The frequency domain characteristics of the acceleration signal are analyzed, and a Fast Fourier transform (FFT) is carried out on the acceleration signal. The power spectrum obtained is shown in Fig 14. Taking node 447492 as an example, the maximum peaks of the four groups are 110.53, 137.11, 210.18, and 214.2. The power spectrum shows the change in signal power with frequency. The area covered by the power spectrum curve is numerically equal to the total energy of the signal. Therefore, the average power spectrum of the four points is calculated, and then, the average value of the four points is calculated. The results are shown in Fig 15. The increase in the gangue content significantly enhances the mean value of the power spectrum, indicating that the energy of the acceleration signal in the frequency domain is also enhanced. This result conforms to Parseval’s theorem, showing that the energies of the signal in the time domain and frequency domain are equal. The discrete form of Parseval’s theorem is

$$\sum_{k=0}^{N-1}{\left|x[k]\right|}^{2}=\frac{1}{N}\sum_{m=0}^{N-1}{\left|X[m]\right|}^{2}$$ (2)

where *x[k]* is the time domain signal or discrete time series, and *X[m]* is the discrete Fourier transform of *x[k]*.

**Fig 16:**
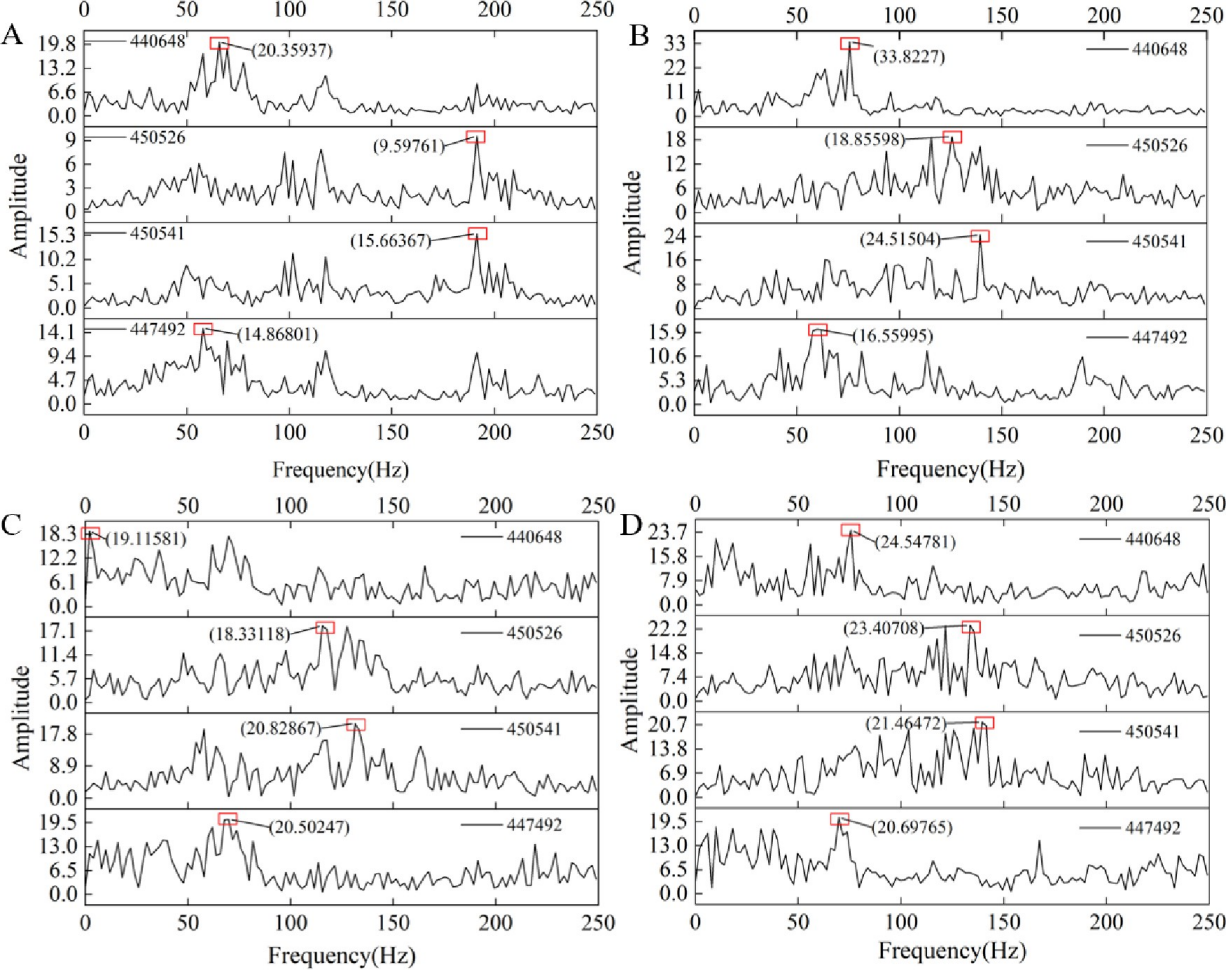
The amplitude spectrum of four nodes with different gangue content. (A) The gangue content of 0%. (B) The gangue content of 25%. (C) The gangue content of 50%. (D) The gangue content of 75%.

**Fig 17:**
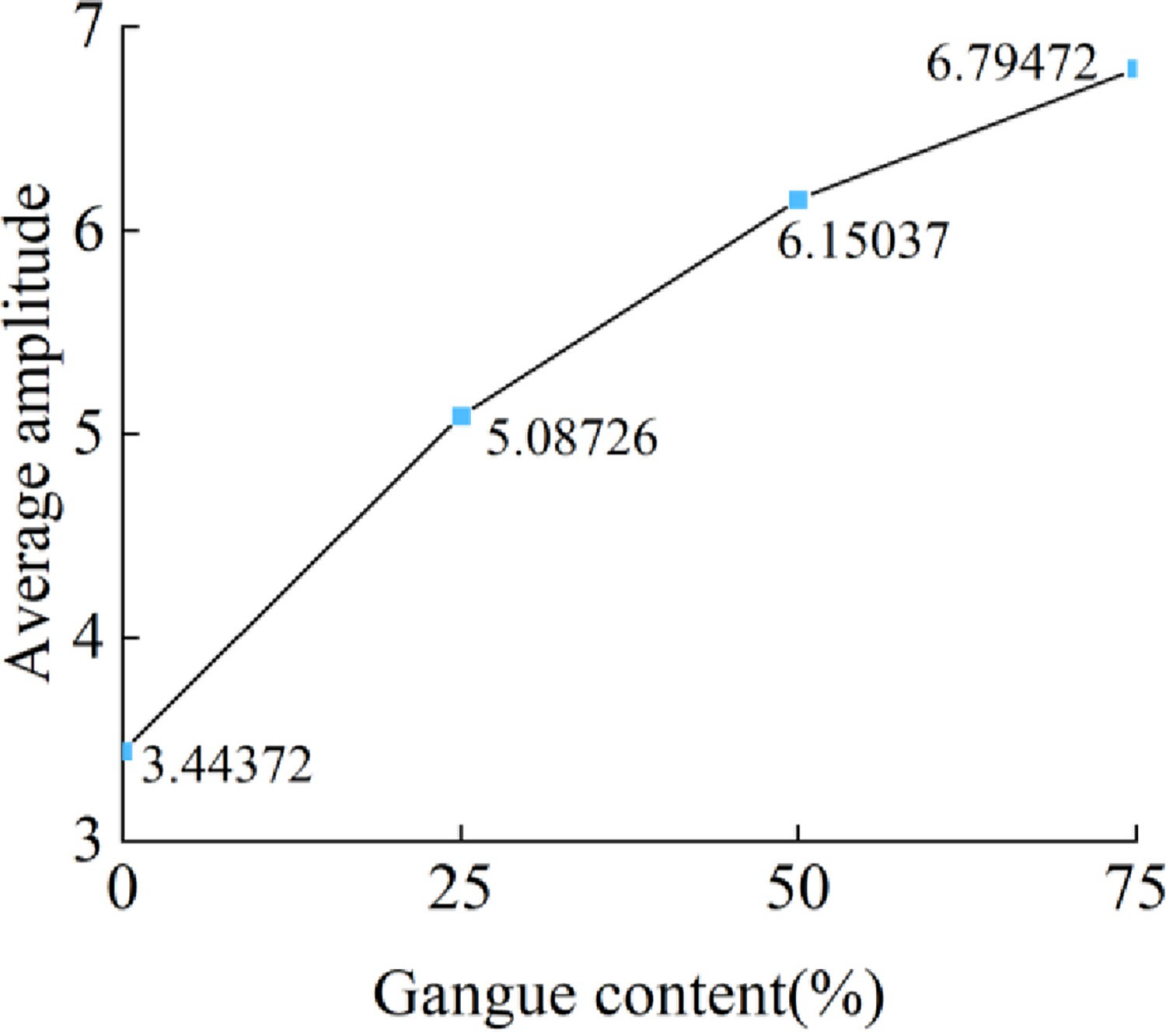
Gangue content versus the average value of the average amplitude.

After obtaining the fast Fourier transform of the acceleration signal, the amplitude spectrum is obtained. The results are shown in Fig 16. Taking node 450526 as an example, with the increase in gangue content, the maximum peaks are 9.5976, 18.856, 18.3312, and 23.4071, and the maximum peak has an upward trend. The average amplitude of each frequency component is calculated, and the results are shown in Fig 17. With the increase in gangue content, the average amplitude shows a significant upward trend.

Different gangue mixing methods may have a certain impact on the results. However, since the judgment results are based on the average value of the four points on the tail beam, we believe that the change in the results may be small, so the coal gangue mixing method is not studies further.

From the sliding test, the increase of in gangue content of the coal gangue particles increases some characteristics of the acceleration signal on the tail beam in the time and frequency domains. Based on the changes in these characteristics, when the gangue content of the coal gangue particles changes along the tail beam, the coal gangue identification method based on vibration has the potential to capture the change in the gangue content, which can provide a reference for the timely closure of the coal opening.

### 3.2 Influence of rock size

**Fig 18:**
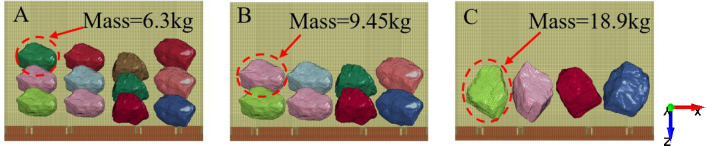
Different rock sizes. (A) The mass of single particle is 6.3kg. (B) The mass of single particle is 9.45kg. (C) The mass of single particle is 18.9kg.

In the process of top coal caving, due to the geological environment, the size of the fragments after the top coal is broken is inconsistent. When the coal strength is high, the number of large coal blocks in caving coal increases. When the coal strength is low, the top coal is usually released in the form of small fragments. Therefore, in this section, the total mass remains unchanged, the number and size of rock particles are changed, and the number of particles is adjusted to 12, 8, and 4. The masses of a single rock are 6.3 kg, 9.45 kg, and 18.9 kg, and the total mass is 75.6 kg. The four gangue contents of 0%, 25%, 50%, and 75% are set for each particle number, and then, the coal gangue particles slide along the tail beam from their static position, with a sliding time of 0.5 s. Fig 18 shows the number and quality of the rock mass.

**Fig 19:**
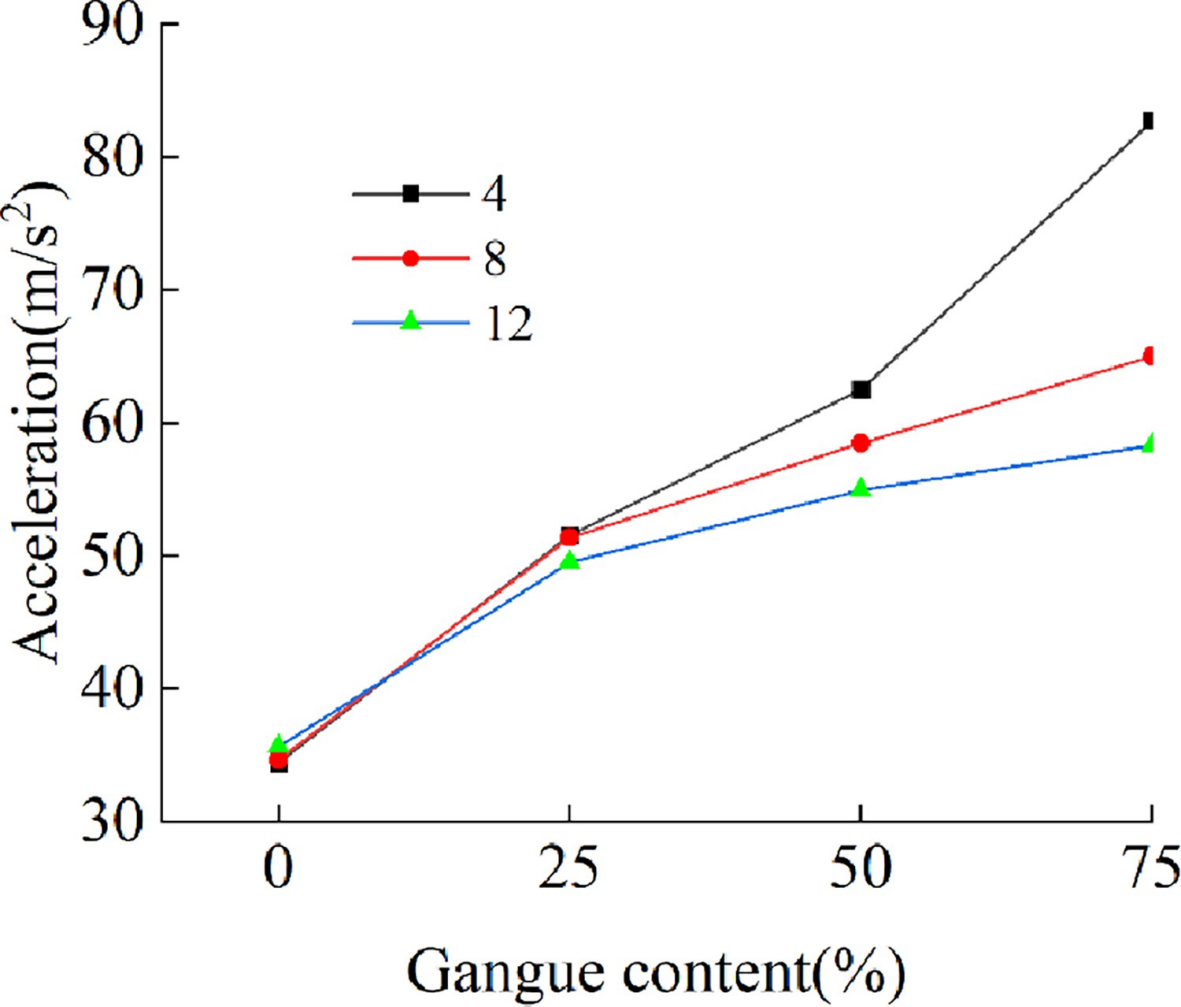
Gangue contents versus acceleration.

Extracting the acceleration signal of the four nodes in Section 3.1, Fig 19 shows the relationship between the gangue content and the acceleration effective value under the condition of different particle numbers. When the numbers of particles are 4, 8, and 12, the acceleration effective value increases with increasing gangue content. When the gangue content is 0%, the acceleration effective value for different particle numbers is approximated, but with the increase in the gangue content, a difference begins to appear. When the gangue contents are 25%, 50%, and 75% and with an increase in gangue content, the acceleration effective values of 4 rocks are 1.04, 1.14, and 1.42 times the acceleration effective value of 12 rocks, respectively. The smaller the number of particles, the higher the intensity of the acceleration signal and the faster the rise. The reason for this may be that when the number of particles is large, the rock mass covers a large area on the tail beam, resulting in an increase in the mass and inertia of the local vibration system at the position covered with the particles, and a weakening of the motion performance of the vibration system.

**Fig 20:**
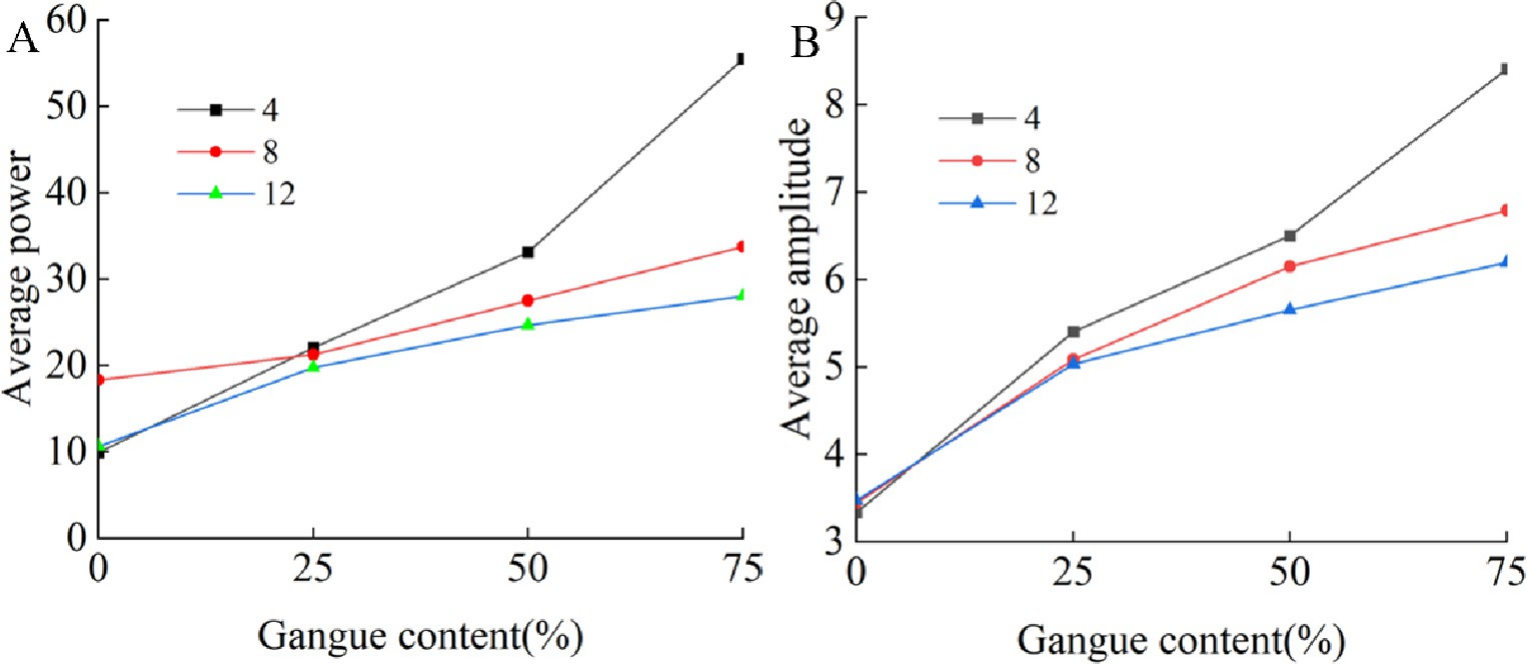
Relationship between gangue content and frequency domain characteristics. (A) Gangue content versus average power under the different rock size. (B) Gangue content versus average amplitude under the different size.

The average power of each test is calculated according to the method in Section 3.1. Fig 20 shows the relationship between the gangue content and the average power and average amplitude for different particle numbers. The variation in the average power and average amplitude is similar to the variation in the acceleration.

For the same total mass, the time and frequency domain characteristics of the acceleration signal on the tail beam caused by the slip of coal gangue particles with different sizes along the tail beam increase with the increase in the gangue content, and when the size of the coal block is larger, the increase in gangue content has a greater impact on the time and frequency domain characteristics. Therefore, when the strength of coal is high and the coal block is large, the coal gangue identification method based on vibration may achieve better results. When the strength of coal is low, some measures should be taken to increase the size of top coal after crushing.

### 3.3 Influence of the total mass of rock mass

**Fig 21:**
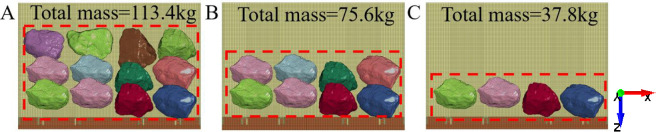
Three coal gangue slipping test with different total mass. (A) The total mass is 113.4kg. (B) The total mass is 75.6kg. (C) The total mass is 37.8kg.

The coal reserves of the mining area and the ratio of mining to caving will affect the thickness of top coal, thus affecting the total mass of the rock mass covering the tail beam. Therefore, this section studies the influence of the total mass of the rock mass on the relationship between gangue content and acceleration. As shown in Fig 21, the numbers of particles in the three groups of tests are 12, 8, and 4, and the total masses of the rock mass are 113.4 kg, 75.6 kg, and 37.8 kg, respectively. For each total mass, tests are carried out on coal gangue particles sliding along the tail beam with gangue contents of 0%, 25%, 50%, and 75%. The time of the numerical simulation is 0.5 s.

**Fig 22:**
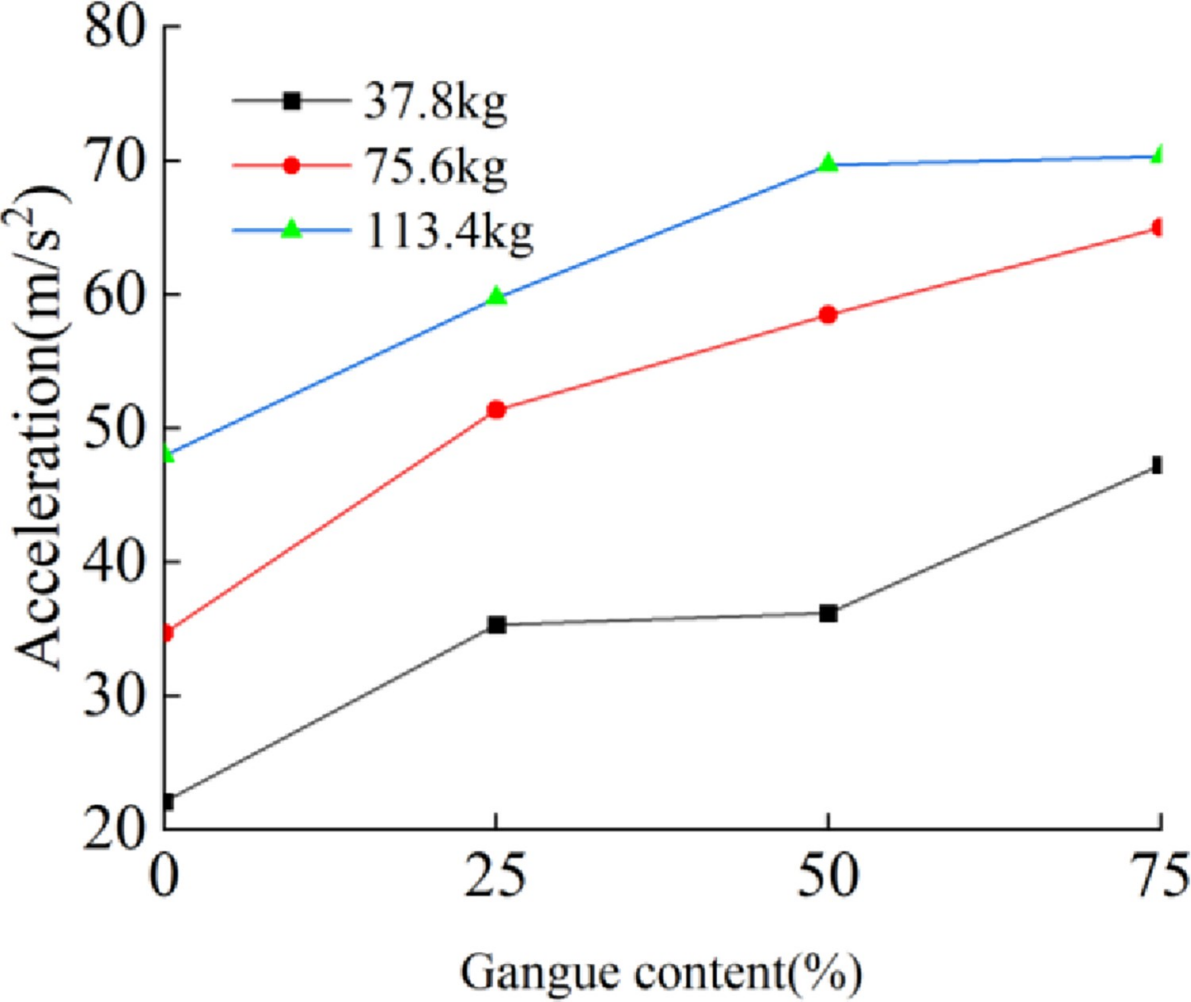
Gangue content versus effective value of acceleration under the mass of 113.4kg, 75.6kg, and 37.8kg.

The acceleration signals of the four nodes on the tail beam are extracted. Fig 22 shows the relationship between the gangue content and the acceleration effective value for different total masses, and the acceleration effective value increases with increasing gangue content. At the same time, the gap between the acceleration effective value for different total masses is obvious. At gangue contents of 0%, 25%, 50%, and 75%, the effective value of acceleration at a total mass of 113.4 kg are 47.96, 59.75, 69.7, and 70.33, respectively, which are larger than the effective value of acceleration at a total mass of 37.8 kg. The increase in mass makes the rock mass transfer more energy to the tail beam. However, the gap between the curves of 12 rocks and 8 rocks is significantly smaller than that between the curves of 8 rocks and 4 rocks. The reason for this may be that the increase in mass increases the mass of the motion system composed of the tail beam and rocks, resulting in an increase in inertia and a weakening of motion performance.

**Fig 23:**
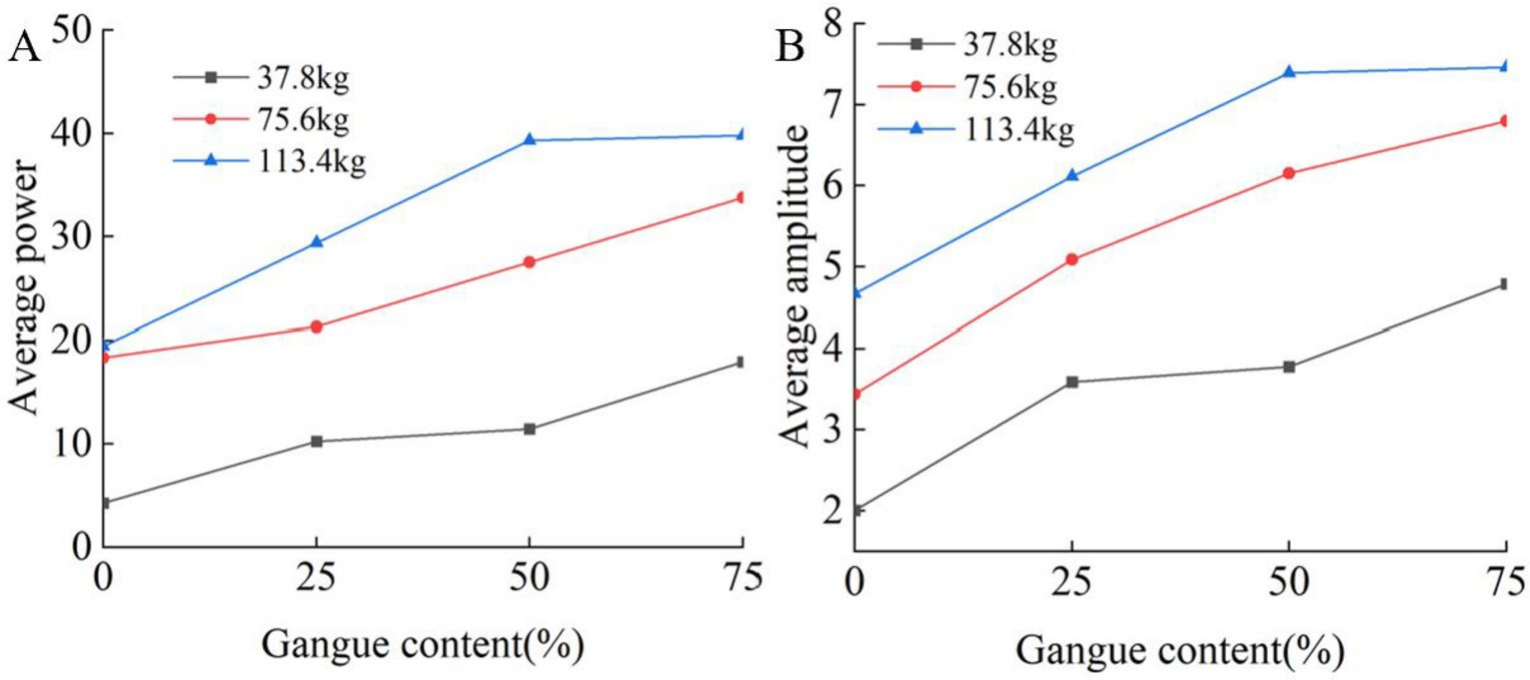
Relationship between gangue content and frequency domain characteristics. (A) Gangue content versus average power and under the total mass of 113.4kg, 75.6kg, and 37.8kg. (B) Gangue content versus average amplitude under the total mass of 113.4kg, 75.6kg, and 37.8kg.

Fig 23 shows the relationship between the average power and average amplitude of the acceleration signal in the frequency domain and the gangue content for different total masses. The results show that the power and amplitude increase with increasing gangue content, and the slope of each curve are similar. Therefore, when some time domain characteristics and frequency domain characteristics of the acceleration signal of the tail beam vibration are used to identify the change in gangue content, the recognition effect is not greatly affected by the total quality of the coal rock on the tail beam. The coal gangue recognition method based on vibration has good adaptability to coal discharge. In the study of total mass, due to the increasing number of particles, the coverage area of coal gangue particles on the tail beam increases. Whether this factor affects the results is not known. In the future, better test methods may be needed to remove the influence of this factor.

### 3.4 Influence of the tail beam angle

In the process of top coal caving, the size of the coal opening refers to the space formed between the tail beam and the goaf. The size of the coal opening can be controlled by the swing angle of the tail beam. The change in the tail beam angle will affect the force of the coal gangue particles in the direction perpendicular to the surface of the tail beam. Therefore, this section simulates the sliding process of the coal gangue particle group along the tail beam with different angles and discusses the influence of the angle of the tail beam on the vibration signal of the tail beam.

**Fig 24:**
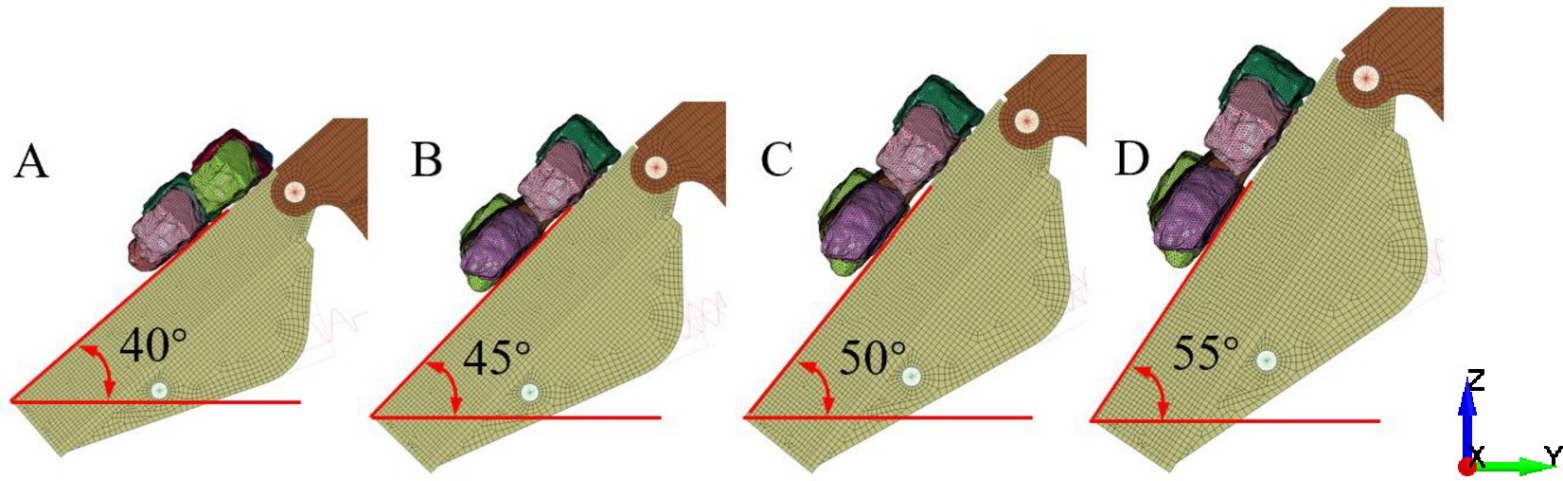
Different tail beam angle. (A) Tail beam angle is 40°. (B) Tail beam angle is 45°. (C) Tail beam angle is 50°. (D) Tail beam angle is 55°.

Fig 24 shows the four angles of the tail beam. The angles between the upper surface of the tail beam and the ground are 40°, 45°, 50°, and 55°. Eight rocks are placed on the tail beam, and they slide from a static state under the action of gravity. The mass of each rock mass is 9.45 kg. At each angle, the sliding process of coal gangue particles with gangue ratios of 0%, 25%, 50%, and 75% along the tail beam are simulated, and the simulation time is 0.5 s.

**Fig 25:**
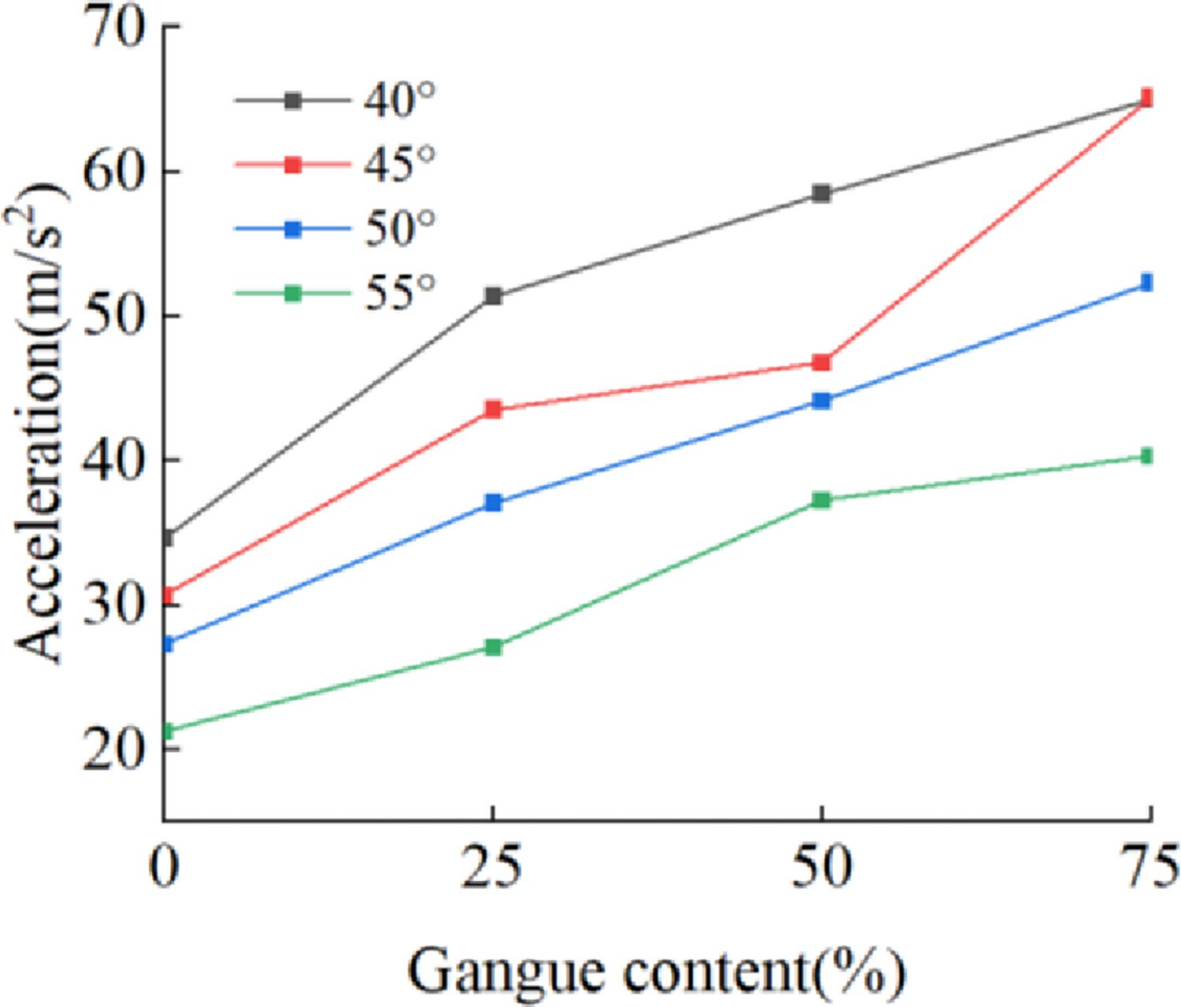
Gangue content versus acceleration under the tail beam angle of 40°,45°,50°,55°.

Extracting the acceleration signal of four nodes, Fig 25 shows the relationship between the acceleration signal and the gangue content at different angles. When the angle between the tail beam and the ground changes from 40° to 55°, the effective value of the acceleration signal increases significantly with increasing gangue content. At the same gangue content, the smaller the angle is, the larger the acceleration effective value because the total mass of the coal gangue particles is the same. When the angle between the tail beam and the ground is smaller, the component of the gravity force of the coal gangue particles in the direction perpendicular to the surface of the tail beam is larger, and the tail beam has a stronger vibration.

**Fig 26:**
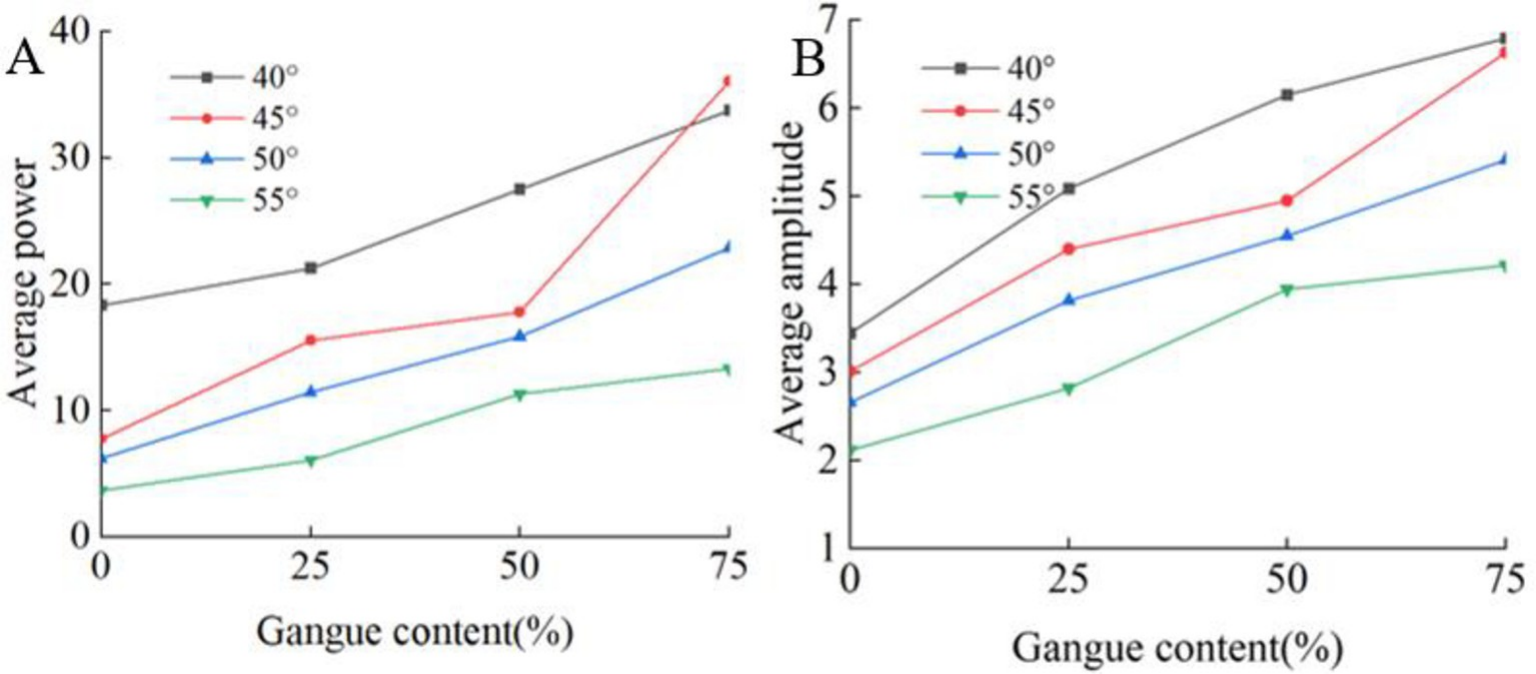
Relationship between gangue content and frequency domain characteristics. (A) Gangue content versus average power under the tail beam angle of 40°,45°,50°,55°. (B) Gangue content versus average amplitude under the tail beam angle of 40°,45°,50°,55°.

Fig 26 shows the relationship between the average power and average amplitude of the acceleration signal in the frequency domain and the gangue content for different tail beam angles. The average power and average amplitude increase with increasing gangue content. Therefore, when the size of the coal discharge port is adjusted by controlling the swing of the tail beam, the change in the gangue content can always cause changes in some of the time and frequency domain characteristics of the acceleration signal on the tail beam, and the smaller the angle between the tail beam and the ground is, the greater the value of these characteristics.

## 4. Conclusions

To prove the identifiability of gangue content when real shape coal gangue particles slide along the tail beam, a finite element model of coal gangue particles and a hydraulic support is established in LS-DYNA. The acceleration signals of four nodes on the tail beam are extracted. The effective value in the time domain, and the average power and amplitude in the frequency domain of the acceleration signal are used as the parameters to evaluate the vibration of the tail beam. The coal gangue identification method based on the real shape and the vibration characteristics of the sliding contact is verified. On this basis, the influence of the size and mass of the rock, and the angle of the tail beam on the signal change trend is analyzed. The feasibility of coal gangue identification based on tail beam vibration characteristics is proven. The following conclusions are drawn:

1. When the coal gangue particles slide along the tail beam, the increase in the gangue content increases the effective value in the time domain, the average amplitude, and the average power in the frequency domain of the tail beam acceleration signal, indicating that some characteristics in the time and frequency domains of the vibration signal of the tail beam can reflect the change in the gangue content. At the same time, a vibration signal with high strength is more likely to appear at the symmetric surface of the tail beam.
2. When the total masses are the same but the numbers and sizes of particles are different, the larger the particle that slips along the tail beam is, the faster the acceleration effective value, average amplitude, and average power rise at different gangue contents. Therefore, the larger the coal gangue is, the greater the difference between different gangue contents.
3. In the case of the same single particle mass, the increase in the total mass of coal gangue particles significantly increases the effective value of acceleration, average power and amplitude. However, due to the increase in the quality of the vibration system, the growth trend weakens.
4. The angle between the tail beam and the ground affects the intensity of the characteristics of the acceleration signal in the time and frequency domains. The smaller the angle between the tail beam and the ground is, the greater the value of the characteristics. However, for different angles, the increase in the gangue content can cause a significant increase in the characteristics of the acceleration signal.

In this paper, the slip process of real shape coal gangue particles along the tail beam is simulated. The results show that it is feasible to judge the change in gangue content by some characteristics of the vibration signal on the tail beam. After testing several factors, it is proven that this method has good adaptability under different conditions. The research results provide a reference for the further study of the coal gangue identification method based on vibration.

Due to the limitation of calculation time and efficiency, the number of coal gangue particles simulated in this paper is still small. In future work, we will continue to find more efficient simulation methods to provide more references for vibration-based coal gangue identification methods.
